# Cardiac biomarkers in patients with renal cell carcinoma treated with immune checkpoint inhibitors

**DOI:** 10.1093/oncolo/oyag079

**Published:** 2026-03-30

**Authors:** Yen-Chou Chen, Mandar A Aras, Brian I Rini, Toni K Choueiri, Paul Cislo, Robert J Motzer, Javid Moslehi

**Affiliations:** Section of Cardio-Oncology and Immunology, Division of Cardiology, and the Cardiovascular Research Institute, University of California, San Francisco, CA 94143, United States; Section of Cardio-Oncology and Immunology, Division of Cardiology, and the Cardiovascular Research Institute, University of California, San Francisco, CA 94143, United States; Department of Medicine, Vanderbilt-Ingram Cancer Center, Nashville, TN 37232, United States; Department of Medical Oncology, Dana-Farber Cancer Institute, Boston, MA 02215, United States; Pfizer, New York, NY 10001, United States; Department of Medicine, Memorial Sloan Kettering Cancer Center, New York, NY 10021, United States; Section of Cardio-Oncology and Immunology, Division of Cardiology, and the Cardiovascular Research Institute, University of California, San Francisco, CA 94143, United States

**Keywords:** avelumab, axitinib, renal cell carcinoma, troponin, B-type natriuretic peptide

## Abstract

Clinical evidence to assess the utility of routine cardiac biomarker surveillance during immune checkpoint inhibitor treatment is limited. We examined cardiac biomarkers at baseline and during initial treatment in the JAVELIN Renal 101 phase 3 trial of avelumab plus axitinib vs sunitinib in patients with previously untreated advanced renal cell carcinoma (NCT02684006). At baseline, among available patients, levels of troponin T, troponin I, B-type natriuretic peptide (BNP), and N-terminal pro-BNP were high (per local assay) in 19.8%, 1.5%, 13.0%, and 32.8%, respectively. In addition, a notable proportion of patients developed high cardiac biomarker levels during treatment in both arms. We did not find an association between elevation in cardiac biomarkers and incidence of major adverse cardiac events, although assessment was limited by the small number of events. Overall, these findings suggest that a high proportion of patients with cancer may have elevated cardiac biomarkers at baseline. Therefore, any attempt at routine surveillance of cardiac biomarkers must include testing of the same biomarker at baseline (before initiating cancer treatment).

## Introduction

Patients treated with immune checkpoint inhibitors (ICIs) may develop immune-related toxicities, including myocarditis, which is associated with high mortality.^1^ Expert consensus statements advocate for assessing cardiac biomarkers to surveil for ICI-related myocarditis.^1^ However, to our knowledge, the diagnostic performance of individual cardiac biomarkers and the prevalence of baseline biomarker abnormalities in patients with cancer have not been fully evaluated in a large clinical trial or “real-world” study.

## Methods

We examined cardiac biomarker levels in 866 patients from the JAVELIN Renal 101 phase 3 trial (NCT02684006), which compared avelumab plus axitinib vs sunitinib in patients with previously untreated advanced renal cell carcinoma.^2^ Levels of troponin T, troponin I, B-type natriuretic peptide (BNP), and N-terminal pro-BNP (NT-proBNP) were measured at baseline and on days 1, 15, and 29 of the first 3 treatment cycles, and levels were categorized as high or not high (including normal or low levels) per local assay cutoff.^2^ Major adverse cardiac events (MACE), including myocarditis, were independently adjudicated using Bonaca criteria.^3^

## Results

**Figure 1:**
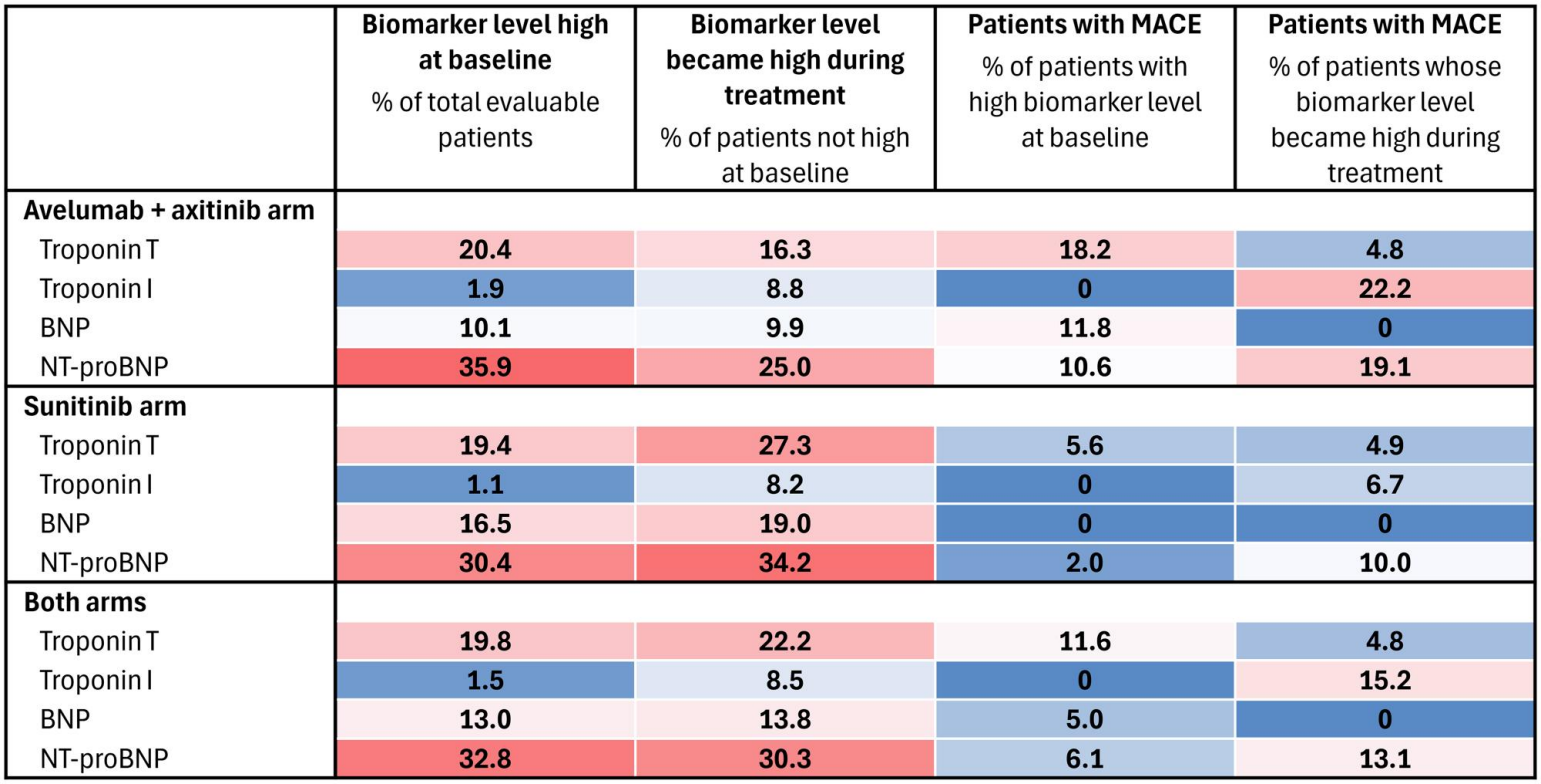
Heat map showing the percentages of patients with high levels of different cardiac biomarkers at baseline or with levels that became high during treatment and the incidence of MACE in both populations. Abbreviations: BNP, B-type natriuretic peptide; MACE, major adverse cardiovascular events; NA, not applicable; NT-proBNP, N-terminal pro–B-type natriuretic peptide.

Among all patients with data available at baseline in both arms, 19.8% (69/348) had high troponin T, 1.5% (6/395) had high troponin I, 13.0% (40/308) had high BNP, and 32.8% (98/299) had high NT-proBNP (Table 1 and Figure 1). Proportions of patients with high cardiac biomarker levels at baseline were similar between study arms. In patients with baseline levels that were not high at baseline, a notable proportion developed high levels after treatment initiation (both arms combined: troponin T, 22.2% [62/279]; troponin I, 8.5% [33/389]; BNP, 13.8% [37/268]; NT-proBNP, 30.3% [61/201]). A higher proportion of patients developed high cardiac biomarker levels during treatment in the sunitinib arm (troponin T, 27.3% [41/150]; BNP, 19.0% [22/116]; NT-proBNP, 34.2% [40/117]) than in the avelumab plus axitinib arm (troponin T, 16.3% [21/129]; BNP, 9.9% [15/152]; NT-proBNP, 25.0% [21/84]).

**Table 1:** Cardiac biomarkers analyzed in patients from the JAVELIN Renal 101 phase 3 trial.

Biomarker	Treatment group (total evaluable patients)	Baseline		On treatment		Patients with high level on treatment, n/N (% of baseline group)	Patients with MACE, n/N (% of on-treatment group) [Fisher’s exact 95% CI]
		Group	Patients, n (% of total evaluable)	Group	Patients, n (% of total evaluable)		
**Troponin T**	Avelumab plus axitinib (*n* = 162)	High	33 (20.4)	High	32 (19.8)	32/33 (97.0)	6/32 (18.8) [7.2-36.4]
				Not high	1 (0.6)		0/1 (0) [0-97.5]
		Not high	129 (79.6)	High	21 (13.0)	21/129 (16.3)	1/21 (4.8) [0.1-23.8]
				Not high	108 (66.6)		5/108 (4.6) [1.5-10.5]
	Sunitinib (*n* = 186)	High	36 (19.4)	High	36 (19.4)	36/36 (100)	2/36 (5.6) [0.7-18.7]
				Not high	0 (0)		NA
		Not high	150 (80.6)	High	41 (22.0)	41/150 (27.3)	2/41 (4.9) [0.6-16.5]
				Not high	109 (58.6)		6/109 (5.5) [2.0-11.6]
	Both arms (*n* = 348)	High	69 (19.8)	High	68 (19.5)	68/69 (98.6)	8/68 (11.8) [5.2-21.9]
				Not high	1 (0.3)		0/1 (0) [0-97.5]
		Not high	279 (80.2)	High	62 (17.8)	62/279 (22.2)	3/62 (4.8) [1.0-13.5]
				Not high	217 (62.4)		11/217 (5.1) [2.6-8.9]
**Troponin I**	Avelumab plus axitinib (*n* = 209)	High	4 (1.9)	High	3 (1.4)	3/4 (75.0)	0/3 (0) [0-70.8]
				Not high	1 (0.5)		0/1 (0) [0-97.5]
		Not high	205 (98.1)	High	18 (8.6)	18/205 (8.8)	4/18 (22.2) [6.4-47.6]
				Not high	187 (89.5)		10/187 (5.4) [2.6-9.6]
	Sunitinib (*n* = 186)	High	2 (1.1)	High	1 (0.5)	1/2 (50.0)	0/1 (0) [0-97.5]
				Not high	1 (0.5)		0/1 (0) [0-97.5]
		Not high	184 (98.9)	High	15 (8.1)	15/184 (8.2)	1/15 (6.7) [0.2-31.9]
				Not high	169 (90.9)		4/169 (2.4) [0.6- 5.9]
	Both arms (*n* = 395)	High	6 (1.5)	High	4 (1.0)	4/6 (66.7)	0/4 (0) [0-60.2]
				Not high	2 (0.5)		0/2 (0) [0-84.1]
		Not high	389 (98.5)	High	33 (8.4)	33/389 (8.5)	5/33 (15.2) [5.1-31.9]
				Not high	356 (90.1)		14/356 (3.9) [2.2-6.5]
**BNP**	Avelumab plus axitinib (*n* = 169)	High	17 (10.1)	High	13 (7.7)	13/17 (76.5)	1/13 (7.7) [0.2-36.0]
				Not high	4 (2.4)		1/4 (25.0) [0.6-80.6]
		Not high	152 (89.9)	High	15 (8.9)	15/152 (9.9)	0/15 (0) [0-21.8]
				Not high	137 (81.1)		9/137 (6.6) [3.0-12.1]
	Sunitinib (*n* = 139)	High	23 (16.5)	High	20 (14.4)	20/23 (87.0)	0/20 (0) [0-16.8]
				Not high	3 (2.2)		0/3 (0) [0-70.8]
		Not high	116 (83.5)	High	22 (15.8)	22/116 (19.0)	0/22 (0) [0-15.4]
				Not high	94 (67.6)		2/94 (2.1) [0.3-7.5]
	Both arms (*n* = 308)	High	40 (13.0)	High	33 (10.7)	33/40 (82.5)	1/33 (3.0) [0.1-15.8]
				Not high	7 (2.3)		1/7 (14.3) [0.4-57.9]
		Not high	268 (87.0)	High	37 (12.0)	37/268 (13.8)	0/37 (0) [0-9.45]
				Not high	231 (75.0)		11/231 (4.8) [2.4-8.4]
**NT-proBNP**	Avelumab plus axitinib (*n* = 131)	High	47 (35.9)	High	46 (35.1)	46/47 (97.9)	5/46 (10.9) [3.6-23.6]
				Not high	1 (0.8)		0/1 (0) [0-97.5]
		Not high	84 (64.1)	High	21 (16.0)	21/84 (25.0)	4/21 (19.1) [5.4-41.9]
				Not high	63 (48.1)		1/63 (1.6) [0-8.5]
	Sunitinib (*n* = 168)	High	51 (30.4)	High	50 (29.8)	50/51 (98.0)	1/50 (2.0) [0.1-10.6]
				Not high	1 (0.6)		0/1 (0) [0-97.5]
		Not high	117 (69.6)	High	40 (23.8)	40/117 (34.2)	4/40 (10.0) [2.8-23.7]
				Not high	77 (45.8)		3/77 (3.9) [0.8-11.0]
	Both arms (*n* = 299)	High	98 (32.8)	High	96 (32.1)	96/98 (98.0)	6/96 (6.3) [2.3-13.1]
				Not high	2 (0.7)		0/2 (0) [0-84.2]
		Not high	201 (67.2)	High	61 (20.4)	61/201 (30.3)	8/61 (13.1) [5.8-24.2]
				Not high	140 (46.8)		4/140 (2.9) [0.8-7.2]

Abbreviations: BNP, B-type natriuretic peptide; MACE, major adverse cardiovascular events; NA, not applicable; NT-proBNP, N-terminal pro–B-type natriuretic peptide.

To investigate the clinical significance of cardiac biomarkers, we examined their association with MACE and myocarditis. In previous post hoc analyses from JAVELIN Renal 101, high troponin T at baseline predicted the risk of MACE with avelumab plus axitinib (relative risk ratio, 3.31; 95% CI, 1.19-9.22).^2^ However, as shown in Table 1, 81.8% of patients (27/33) in the avelumab plus axitinib arm who had a high troponin T level at baseline did not develop MACE. The overall incidence of MACE among all evaluable patients in this arm was 7.4% (12/162), suggesting that using baseline troponin T as a predictor for MACE may result in a high false-positive rate. For most cardiac biomarkers, in patients whose levels were not high at baseline but became high during treatment, the incidence of MACE was similar in the avelumab plus axitinib and sunitinib arms (troponin T, 4.8% [1/21] and 4.9% [2/41]; troponin I, 22.2% [4/18] and 6.7% [1/15]; BNP, 0% [0/15] and 0% [0/22]; NT-proBNP, 19.1% [4/21] and 10.0% [4/40]; respectively), with overlapping 95% CIs (Table 1 and Figure 1). Early elevations in troponin T, troponin I, BNP, and NT-proBNP were not significantly associated with MACE, given that patients whose biomarker levels remained not high had similar MACE incidence between arms. Assessment of myocarditis was limited because only 7 patients had definite, probable, or possible myocarditis (diagnosed or suspected by magnetic resonance imaging or clinical syndromes); of these patients, 3 had normal troponin T or troponin I levels during treatment.^2^

## Discussion

Our study prospectively assessed cardiac biomarkers at baseline and during follow-up in a trial comparing frontline ICI-based combination treatment vs non-ICI treatment. A notable proportion of patients had high cardiac biomarkers at baseline and during follow-up. However, our results did not show an association with cardiac events (either MACE or myocarditis). A previous prospective study similarly identified a lack of association when using troponin I for routine surveillance of ICI-related myocarditis; 11.2% of patients (24/214) had abnormal troponin I during treatment, but only 1.4% (3/214) had ICI-related myocarditis, resulting in a positive predictive value of 12.5%.^4^ In our analysis, troponin T was elevated more frequently than troponin I (131/348 [37.6%] vs 39/393 [9.9%]), but the prevalence of MACE in elevated subgroups was similar (troponin T, 22/348 [6.3%]; troponin I, 19/393 [4.8%]). Although troponin I is considered more cardiac-specific, troponin T has been reported to be more sensitive for detecting ICI-related myocarditis—likely because it may rise in patients with concurrent ICI-related myositis.^5^ Findings from our study suggest that the interpretation of troponin or natriuretic peptide levels is challenging. These observations underscore the need for further assay-based studies to clarify potential differences in diagnostic and prognostic performance, particularly between troponin T and troponin I, in the setting of ICI-related myocarditis. Furthermore, because a higher incidence of elevated troponin or natriuretic peptide is seen at baseline in patients with cancer who have chronic kidney disease, preexisting cardiovascular diseases, sustained tachycardia, or previous anticancer therapy (eg, anthracyclines), interpretation of cardiac biomarker levels may be more challenging.^6^^,^^7^ Baseline measurements help to understand the clinical significance of subsequent rises. Given the potential risk for MACE with preexisting cardiovascular disease, management of patients with elevated baseline cardiac biomarkers should include early involvement of cardiologists and close monitoring, including symptom assessment and electrocardiography. In patients who develop new cardiac symptoms or new electrocardiographic abnormalities, repeat cardiac biomarker testing and echocardiography should be considered promptly.^8^ Our study is limited by the use of local thresholds to define high biomarker levels, lack of patient-level absolute troponin values, and unavailable mortality data, which limit assessment beyond MACE. In practice, however, any recommendation regarding biomarker surveillance for myocarditis would rely on local thresholds.

Overall, the low specificity of conventional cardiac biomarkers during follow-up may lead to unnecessary diagnostic cardiac testing and treatment interruption, emphasizing the need for more specific biomarkers to identify patients at higher risk of myocarditis. Currently, reported risk factors for developing myocarditis include combination ICI treatment (ie, anti-PD-[L]1 plus anti-CTLA-4 or anti-LAG3) and thymoma/thymic cancer.^9^^,^^10^ The existence of high cardiac biomarker levels at baseline underscores the need to perform baseline testing before ICI treatment if cardiac markers are used in routine surveillance.

## Data Availability

Upon request, and subject to review, Pfizer will provide the data that support the findings of this study. Subject to certain criteria, conditions, and exceptions, Pfizer may also provide access to the related individual deidentified participant data. See https://www.pfizer.com/science/clinical-trials/trial-data-and-results for more information.
